# Molecular mechanism of brassinosteroids involved in root gravity response based on transcriptome analysis

**DOI:** 10.1186/s12870-024-05174-6

**Published:** 2024-06-01

**Authors:** Qunwei Bai, Shurong Xuan, Wenjuan Li, Khawar Ali, Bowen Zheng, Hongyan Ren

**Affiliations:** 1 College of Life Sciences, Shaanxi Normal University, Xi’an, Shaanxi Province 710119 PR China; 2 Shaanxi Key Laboratory of Chinese Jujube, College of Life Sciences, Yan’an University, Yan’an, Shaanxi Province 716000 PR China

**Keywords:** Brassinosteroids, DET2, Gravitropism, Phytohormones

## Abstract

**Background:**

Brassinosteroids (BRs) are a class of phytohormones that regulate a wide range of developmental processes in plants. BR-associated mutants display impaired growth and response to developmental and environmental stimuli.

**Results:**

Here, we found that a BR-deficient mutant *det2-1* displayed abnormal root gravitropic growth in Arabidopsis, which was not present in other BR mutants. To further elucidate the role of DET2 in gravity, we performed transcriptome sequencing and analysis of *det2-1* and *bri1-116, bri1* null mutant allele. Expression levels of auxin, gibberellin, cytokinin, and other related genes in the two mutants of *det2-1* and *bri1-116* were basically the same. However, we only found that a large number of JAZ (JASMONATE ZIM-domain) genes and jasmonate synthesis-related genes were upregulated in *det2-1* mutant, suggesting increased levels of endogenous JA.

**Conclusions:**

Our results also suggested that DET2 not only plays a role in BR synthesis but may also be involved in JA regulation. Our study provides a new insight into the molecular mechanism of BRs on the root gravitropism.

**Supplementary Information:**

The online version contains supplementary material available at 10.1186/s12870-024-05174-6.

## Introduction

Gravity is an important environmental cue that guides plant organ growth. Plants are able to orient growth to gravity, ultimately controlling the architecture of the shoot and root [1–4]. Generally, the plant gravity response involves three steps: gravity sensing, signal transduction and transmission, and finally growth regulation. A series of Arabidopsis studies have demonstrated that gravity perception is mediated by amyloplasts sedimentation in the root cap columella cells [5, 6]. Once the plant senses the gravity field, sediment and repositioning of amyloplasts are triggered. This may alter the plasma membrane tension, thereby activating mechano-sensitive ion channels, resulting in transient changes in Ca^2+^ fluxes and triggering gravity signal transduction [7–9]. Previous studies have shown that Arabidopsis starch-deficient mutants *pgm1* exhibited an abnormal gravity response [10, 11], while excess starch mutants *sex1* display an increased sensitivity to gravistimulation [12]. In addition, proton flow can also be used as the second messenger of gravity signal transduction. Rapid changes in the cytosolic PH of the columella cells can be detected within 2 min of receiving the gravistimulation [13, 14]. These changes may contribute to establish a polarity of the root-cap columella cells. A small number of genes involved in the early gravity signal transduction phases have also been identified. *ARG1* (*AlteredResponse toGravity 1*) which encodes a DnaJ-like protein appears to be one of them. Mutation in this gene affected root and hypocotyl gravitropism without pleiotropic phenotypes [15, 16]. This peripheral membrane protein has been implicated as a molecular chaperone mediating the other protein folding, activity, or interactions with variety protein substrates [7, 17]. Similarly, the *ARG1-Like 2 (ARL2)* was also found to play a role in gravitropism and mutations in *ARL2* displayed a similar phenotype to that of ARG1. Although the mutant phenotypic effects of ARL2 were weaker than ARG1, both, arguably function on the same signal pathway [18]. Some studies suggested that ARG1 and ARL2 are required for the auxin redistribution, at least in part by regulating the localization of the auxin efflux carrier PIN3 [15, 19].

Auxin is the primary hormone involved in gravitropic response which mediates gravity induced cell differential elongation [20, 21]. Many auxin response mutants are reported showing defects in gravitropism [22, 23]. Once the gravity signal pathway is activated, asymmetric auxin distribution follows. This requires auxin influx and efflux carriers of the AUX/LAX and PIN protein families, respectively [17, 23–25]. During this process the auxin is transported to the lower side of the root elongation area, then promoting the downward bending of the root tip. Alteration of the cellular auxin efflux is mainly determined by PIN proteins, in particular PIN1, PIN2, PIN3 and PIN7. PIN3 and PIN7 initiate the root gravitropism in the columella [26–28]. These two proteins have similar expression patterns and are functionally redundant. When the root is upright, PIN3 and PIN7 are symmetrically localized on the root columella cell plasmalemma, however, after gravity sensing, both proteins quickly localize on the bottom side of the root columella cells, and mediate the auxin flux toward the lower side [26, 27]. Subsequently, auxin will be transported further by AUX1 influx and PIN2 efflux carriers from the columella cells to the elongation zone epidermal cells [29, 30], where ARF7 and ARF19 are believed to trigger the auxin response pathway and ultimately inhibited cell elongation on the lower side of the root [31].

In addition to auxin, other hormones are also involved in the gravitropic response. Since the 1970s gibberellic acid (GA) has been implicated in gravity responses [32]. In gravitropic response, GA shows a similar asymmetric distribution as auxin, and the maximum distribution is observed on the lower side of roots [33–35]. GA could be involved in regulating and stabilizing auxin efflux carriers. GA localization is related to the localization of the PIN2 protein. GA can inhibit PIN2 proteins trafficking to the vacuole for degradation, and increase PIN2 protein abundance on the plasma membrane [36]. Cytokinin (CK) usually has a negative regulatory effect on root growth, and on the root gravitropism. During the early stage of gravitropic response, CK symmetric expression pattern in the vertical root cap rapidly changes to the asymmetric expression pattern with a high concentration on the lower side of the root cap [37]. Another phytohormone, ethylene may also play an important role in the gravitropic response, however, its effect on this process has not been well-established [38, 39]. Some suggested that ethylene displays a positive response to gravity [40–42]. Gravity can obviously induce ethylene production in the lower side of the root elongation zone, which may result from increased free IAA levels [43]. However, others suggest that ethylene can reduce starch levels in the columella cells, thereby inhibiting the root gravitropic response [44, 45]. Since amyloplasts are considered necessary for gravity sensing, these results also imply that ethylene may be involved in the gravity sensing stage. Jasmonates (JA) are also known to form a gradient opposite to the auxin gradient, which may positively regulate gravity bending, and JA-gradient formation is independent of the IAA-gradient [46]. Brassinosteroids (BRs) are plant steroid hormones that regulate almost plant growth and development stages, and are involved in plant gravitropic response [47, 48]. BR-dependent regulation is related to auxin gravitropic responses [48–51]. Exogenous application of brassinolide (BL) is known to enhance maize primary roots gravitropic responses [52], tomato hypocotyls [53], or rice lamina joints [54], and is more prominent in the presence of IAA, but weakened in the presence of auxin transport inhibitors NPA and TIBA [55]. Some research suggests that BL may govern PIN2 transverse gradient formation by controlling PIN2 endocytic sorting after gravity stimulation. Although the effect of BL on plant gravitropism is obvious, understanding its molecular regulation mechanism is still limited [48, 49].

DET2 encodes a steroid 5α-reductase which catalyzes a major rate-limiting step in BR biosynthesis. The *det2-1* mutant shows a reduced endogenous BR accumulation and displays a dwarf phenotype and defects in root and leaf development [56]. *bri1-116* is a null-mutant resulting from a premature stop codon insertion at position 583 of the BRI1 receptor displaying a severe dwarf phenotype, shortened petioles, and shrunken and rounded leaves, and the endogenous BR increased in *bri1-116* [57]. Here, we found that *det2-1* shows a loss gravity phenotype, which was not present in other BR mutants, such as, *bri1-116*, *bri1-301*, *cpd*, and *dwf4*. To understand the differences between *det2-1* mutant and other BR mutants on root gravitropism, we choose the null mutant of BR receptor, *bri1-116* and *det2-1* mutant for further study. we studied the root morphological characteristics of them, and analyzed their root transcriptome. Transcriptome analysis showed that multiple genes related to plant hormone response were significantly changed in the two mutants, and the number of differentially expressed genes (DEG) in the *det2-1* mutant was significantly greater than the *bri1-116* mutant, which corresponds to the more obvious phenotype of *det2-1* phenotype. JA-associated DEGs in *det2-1* are the most prominent, indicating their special relationship with the *det2-1* mutant.

## Materials and methods

### Plant materials and growth conditions

The Arabidopsis thaliana Columbia (Col-0) was used as wild type (WT) in this study. *bri1-116* (*brassinosteroid insensitive 1-116*) and *det2-1* (*de-etiolated-2-1*) mutants are in Col-0 background which have been described in previous studies [56, 57]. Seeds were germinated on 1/2 Murashige & Skoog (MS). For *det2-1* and *bri1-116* genotyping, the genomic DNA regions adjacent to the mutation sites were amplified and then digested with Mn1I and PmeI, respectively. The restriction sites were lost in *det2-1* and *bri1-116*, respectively, so DNA fragments with different sizes can be distinguished after electrophoresis. Wild type Col-0 was used as control. Primers used in this study were given in Table S4.

### cDNA library construction

5 g root sample were collected from 7-day-old Col-0, *bri1-116* and *det2-1* seedings. For each sample, about 10 µg total RNA was extracted using trizol reagent (Invitrogen, Shanghai, China) according to its operation manual. RNA integrity was determined by a Bioanalyzer 2100, and RNA with an integrity number > 7.0 was used for further study. For target mRNA purification, oligo-dT magnetic beads were used to isolate mRNA with polyA. Then, the target RNA was fragmented and reverse transcribed to cDNA by a series of random primers. The ligation products were amplified by two specific primers and denatured to produce single strand cDNA. At last, the single strand cDNA was cyclized by DNA ligase for library preparation.

### Constructs and transgenic plants generation

The cDNA sequences of *DET2* were introduced into *p35S:: GFP*. The *p35S:: DET2-GFP* plasmid was transformed into *det2-1*. The transformants were screened on 1/2 MS with 40 µg/ml kanamycin. Primers used in this study were given in Table S4.

### Differentially expressed genes (DEGs) screening

To screen DEGs in different mutants, we used the reads per kb per million reads (RPKM) method to measure the gene expression levels. According to the different RPKM values of the expressed genes in the different mutants, the screening parameters for DEGs were set as follows: p value < 0.05 and Log2 (Fold Change) ≥ 2. According to the RPKM value of each gene in the mutant, cluster analysis and differential gene expression profiling were performed.

### Clustering analysis of expression pattern

Clustering analysis of expression patterns was performed with the K-means method. Each column represents the different plants, and each row represents a gene. Log2 (fold change) values were used to show the differential expression. Blue and purple boxes represent genes showing lower and higher expression levels, respectively.

### Enrichment analysis of GO enrichment and KEGG pathway

The annotation function of GO analysis is comprised of three categories: BP, CC, and MF. Kyoto Encyclopedia of Genes and Genomes (KEGG) is a database resource for understanding high-level functions and utilities of genes or proteins. GO analysis and KEGG pathway enrichment analysis of candidate DEGs were performed using the R package.

### Phenotypic analysis

Root hair was captured using a stereoscope with CCD. The number and length of root hairs were measured in the root hair differentiation zone or a selected portion (0.5 mm long) of this region using ImageJ software (http://imagej.nih.gov). Root angles were measured and placed into one of the 6 bins covering 360°, set at 60° intervals. Distribution of the root gravitropic angle in the plant within 6 bins.

### Starch staining

Five-day-old seedlings were stained in 10%/5% KI/I solution. Stained roots were cleared with chloral hydrate prior to observation under the microscope with a 40× objective.

### qRT-PCR analysis

Total RNA was isolated from roots of Col-0, *bri1-116* and *det2-1* using a HiPure Plant RNA Mini Kit (Magen, R4151-02) according to the protocol provided by the manufacturer. First-strand cDNA was synthesized from 2 µg of total RNA using HiScript II Q RT SuperMix (Vazyme, R223-01). The qRT-PCR was performed using ChamQ SYBR qPCR Master Mix (Vazyme, Q311) to detect the transcript levels of genes. *ACTIN2* (*ACT2*) was used as an internal control. The primers used for qRT-PCR are listed in Table S4.

### Statistical analysis

Statistical analysis was performed using two-way ANOVA with Sidak’s test, as implemented in GraphPad Prism 8.0. (GraphPad Software, http://www.graphpad.com*).*

## Results

### The *det2-1* mutant displays a pronounced loss of root gravitropism

**Fig. 1:**
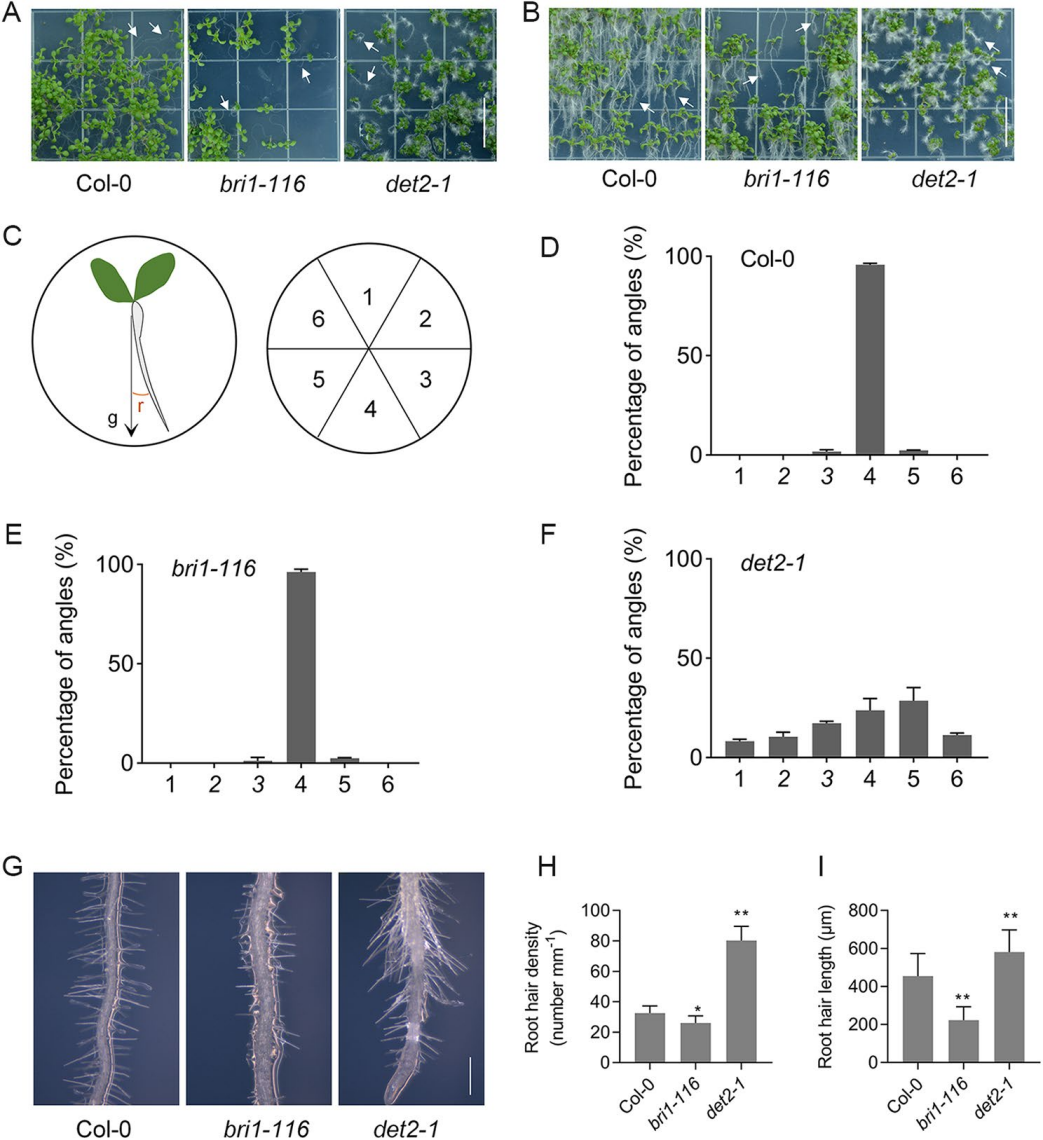
*det2-1* roots grow in random directions with denser and longer root hairs. (**A**) The 7-day-old seedlings were grown horizontally in 1/2 MS medium. The white arrow indicates the root of the seedling. Scale bar, 1.5 cm. (**B**) The 7-day-old seedlings were grown vertically in 1/2 MS medium. The white arrow indicates the root of the seedling. Scale bar, 1.5 cm. (**C**) Schematic diagram showing measurement of the root gravitropic angle (r). Root angles relative to the gravity vector (r) were measured and placed into one of the 6 bins, set at 60° intervals. (**D-F**) Distribution of the root gravitropic angle in Col-0, *bri1-116* and *det2-1* within 6 bins covering 360°. (**G**) Root hair phenotypes of Col-0, *bri1-116* and *det2-1*. Scale bar: 500 μm. (**H**) Quantitative analysis of root hair length of G. (**I**) Root hair number, measured within 500 μm of the root length. *P < 0.01, **P < 0.001 (one-way ANOVA with a Tukey’s test)

To investigate how BRs are involved in plant root gravitropism regulation, we investigated the gravitropic responses of BR biosynthesis mutant *det2-1* and BR insensitive mutant *bri1-116*, and found that *det2-1* mutant roots did not penetrate into the medium, but grew in the air and on the media surface with denser and longer root hairs (Fig. 1A, B). We measured and compared the roots angles of the Col-0, *bri1-116*, and *det2-1* plants and found that *det2-1* mutant roots grow in random directions (Fig. 1B-F). In addition, overexpression of *DET2* could repress the abnormal root gravity phenotype of the *det2-1* mutant (Figure S1). Next, we measured the root hair density and root hair length of 7-day-old seedlings and found that the *bri1-116* and *det2-1* mutants displayed different phenotypes. Root hair density and length of root hair were significantly reduced in the *bri1-116* mutant, but increased significantly in the *det2-1* mutant (Fig. 1G-H).

We then analyzed the expression of plant gravitropism-related genes to check the expression difference. *ARG1* encodes the J-domain protein located in endomembrane organelles, which acts on the root statocytes to facilitate gravitropism. We found that the expression of *ARG1* was significantly down-regulated in both *bri1-116* and *det2-1* mutants, especially in *det2-1* mutants (Figure S2 C). Similarly, the expression of another gene, *PGM1* which encodes phosphoglucomutase, is involved in statoliths synthesis, and plays a role in gravitropism was significantly down-regulated in both, *bri1-116* and *det2-1* mutants. (Figure S2 B). Amyloplasts distribution in the root cap columella cells was also changed in both mutants, especially in the *det2-1* mutants, where amyloplasts were inconspicuously scattered in root tip and cap cells (Figure S2 A). Overall, these results showed that BRs were involved in Arabidopsis roots gravitropism response, and the *det2-1* mutant showed a stronger gravitropism loss than the *bri1-116* mutant.

### More differentially expressed genes were detected in the* det2-1* than in the *bri1-116* mutant

**Fig. 2:**
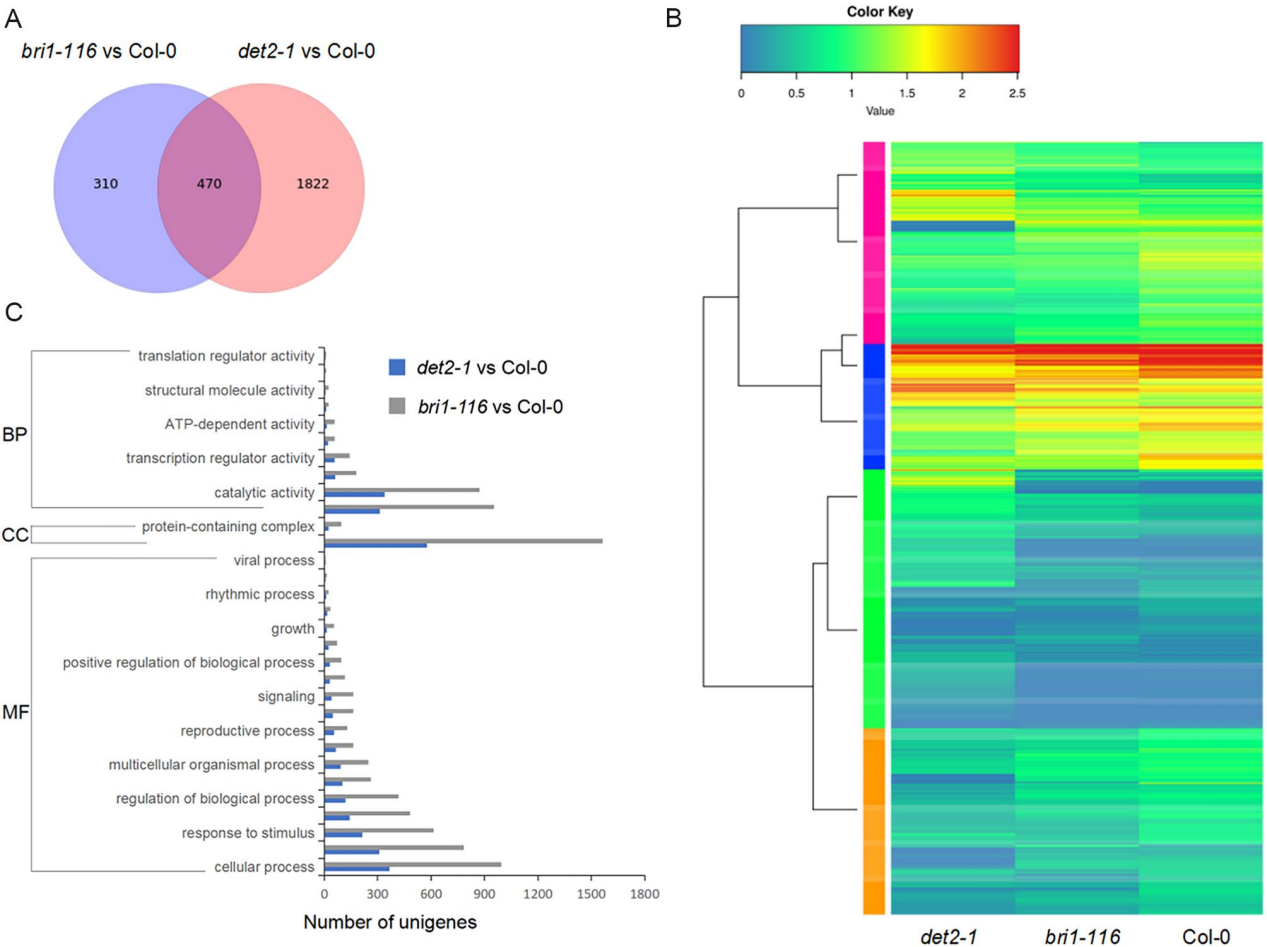
Transcriptional variation in *det2-1* and *bri1-116* mutants. (**A**) Venn diagrams of the regulated DEGs in different comparisons. (**B**) Expression profiles of the differential expressed unigenes in *det2-1*, *bri1-116* and Col-0. Horizontal rows represent individual unigenes, and vertical columns represent each sampled point. Log 2 -transformed fold-change values were used for eachunigene at each infection stage. As shown on the color scale at the bottom of the figure, red indicates up-regulated genes and blue indicates down-regulated genes. (**C**) GO function classifications of unigenes for *det2-1* and *bri1-116* compared with Col-0. BP, biological process; CC, cellular component; MF, molecular function.

**Fig. 3:**
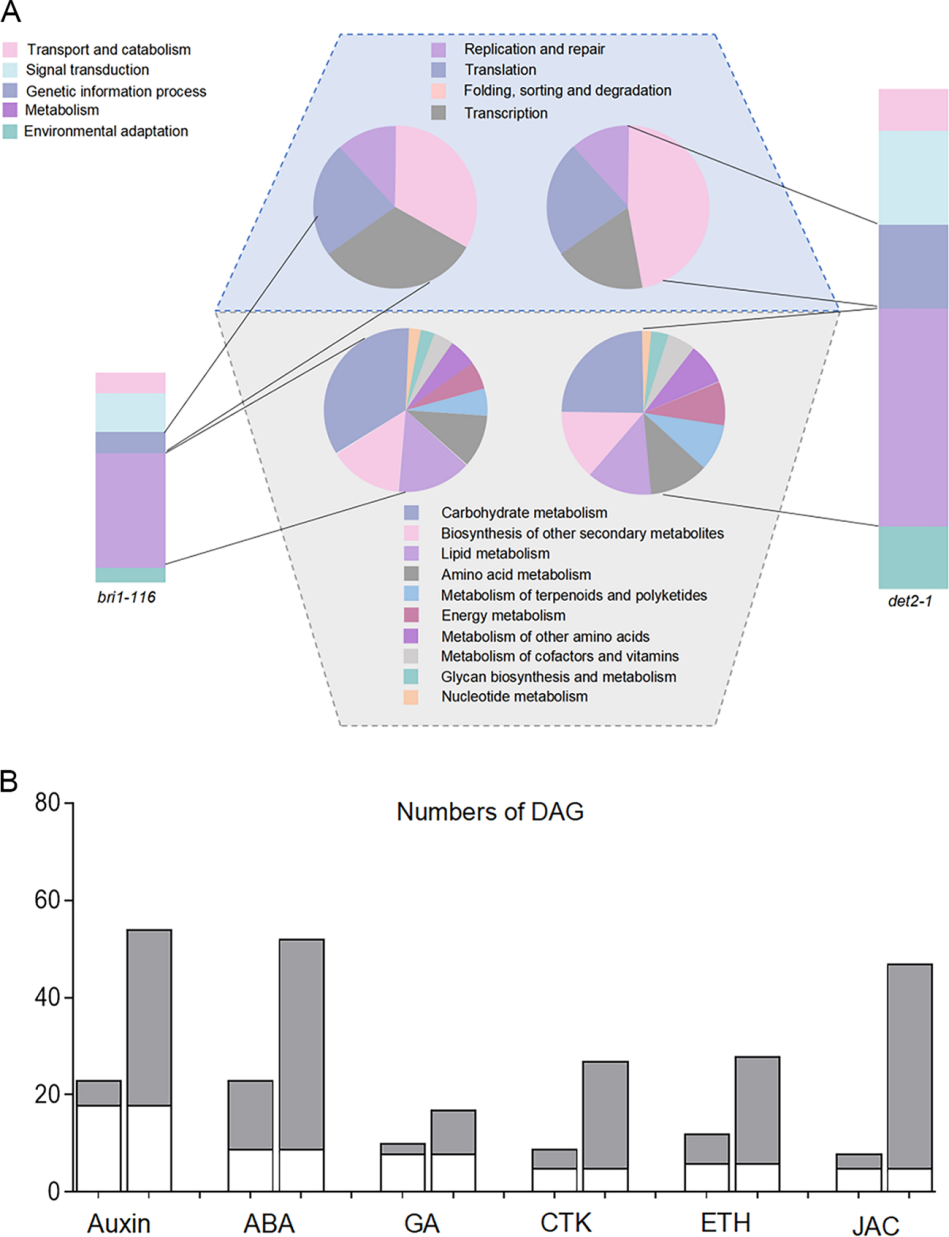
Classification of enriched KEGG terms of the DEGs. (**A**) All the DEGs were classified into different KEGG terms. (**B**) The number of DEGs involved in various hormonal signaling pathways. The first column represents the DEGs in *bri1-116*, while the second column represents the DEGs in *det2-1*, the ABA, abscisic acid; GA, Gibberellic acid; CTK, cytokinin; ETH, cytokinin; JA, jasmonic acid

To identify the main differences between *det2-1* and *bri1-116* mutant DEGs responsible for root development, we analyzed the Go terms and the KEGG terms of the DEGs. Most DEGs could be assigned into 31 major GO terms, such as “cellular anatomical entity”, “cellular process”, “binding”, “catalytic activity”, “metabolic process”, and “response to stimulus” terms (Fig. 2C). Then, the DEGs were mapped to the reference canonical pathways in the KEGG database to identify different pathways between these two mutants. 373 DEGs of *det2-1* mutant were significantly enriched into 27 different pathways (Table S2, Fig. 3A), and 116 DEGs of *bri1-116* mutant were classified into 13 pathways (Table S3 Fig. 3A). For both mutants, the most enriched KEGG pathways were the “Plant hormone signal transduction”, “Phenylpropanoid biosynthesis”, “MAPK signaling pathway plant”, and “Starch and sucrose metabolism”. The DEGs of the *det2-1* mutant were associated with more KEGG pathways which indicated that the *det2-1* mutant may involve more signaling pathways than the *bri1-116* mutant (Figure S4). Overall, it appears that Arabidopsis roots may adapt to BR deficiency through phytohormone regulation and carbohydrate metabolism, while *DET2* may play a role in more signaling pathways or metabolic pathways than *BRI1* (Fig. 3B).

### Identification of auxin-related DEGs

**Fig. 4:**
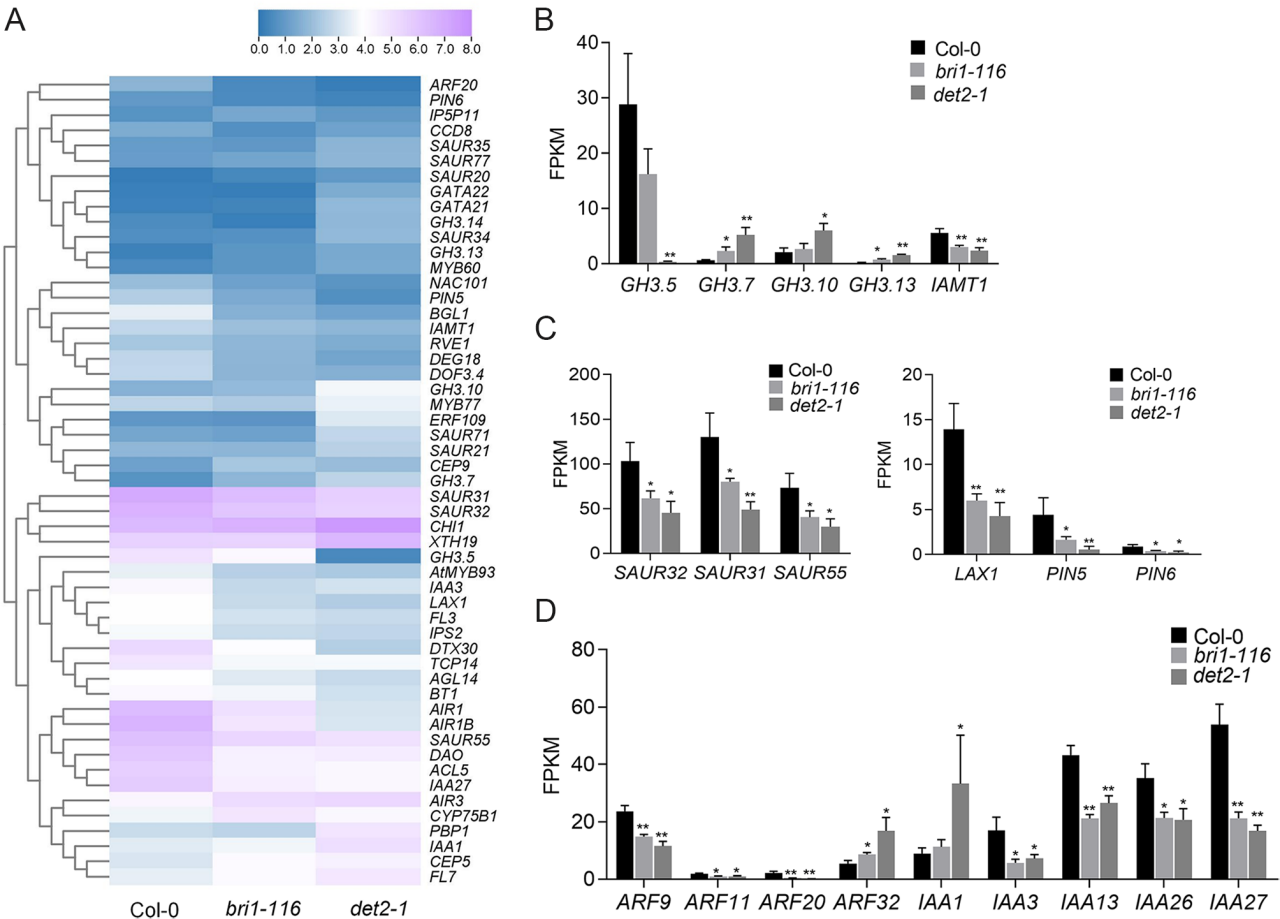
Auxin-related DEGs (**A**) Relative heat maps of RNA-seq. Rows represent auxin related genes, columns represent Col-0, *bri1-116* and *det2-1*. Blue and purple boxes represent genes showing lower and higher expression levels, respectively. Color saturation reflects the magnitude of the expression level for each gene. (**B-D**) Comparison of the auxin-related gene expression levels (by FPKM values) in Col-0, *bri1-116*, and det2-1. *P < 0.05, **P < 0.01 (t-test)

The polar transport of auxin is a driver of root gravitropism; thus, we subsequently analyzed different auxin-related genes expression in *bri1-116* and *det2-1* mutants. From our expression analysis we found that the expression of *GH3.5* and *IAMT1* were down-regulated in both *bri1-116* and *det2-1* mutants. The *GH3.5* encodes an IAA-amino synthase that conjugates amino acids to auxin, while *IAMT1*, encodes an IAA methyltransferase 1 that converts IAA to its methylester form MeIAA. Similarly, the auxin influx carriers *LAX1* and efflux carriers *PIN5* and *PIN6* were also down-regulated. Some SAUR family members, such as *SAUR32*, *SAUR31* and *SAUR55*, were down-regulated to varying degrees, and some auxin response factors were also significantly changed. Among the *IAA* family, *IAA3*, *IAA13*, *IAA26* and *IAA27* were significantly down-regulated, while IAA1 was up-regulated, and among the *ARF* family, *ARF9, ARF11* and *ARF20* were significantly down-regulated, whereas *ARF32* was up-regulated (Fig. 4).

### Identification of cytokinin-related DEGs

**Fig. 5:**
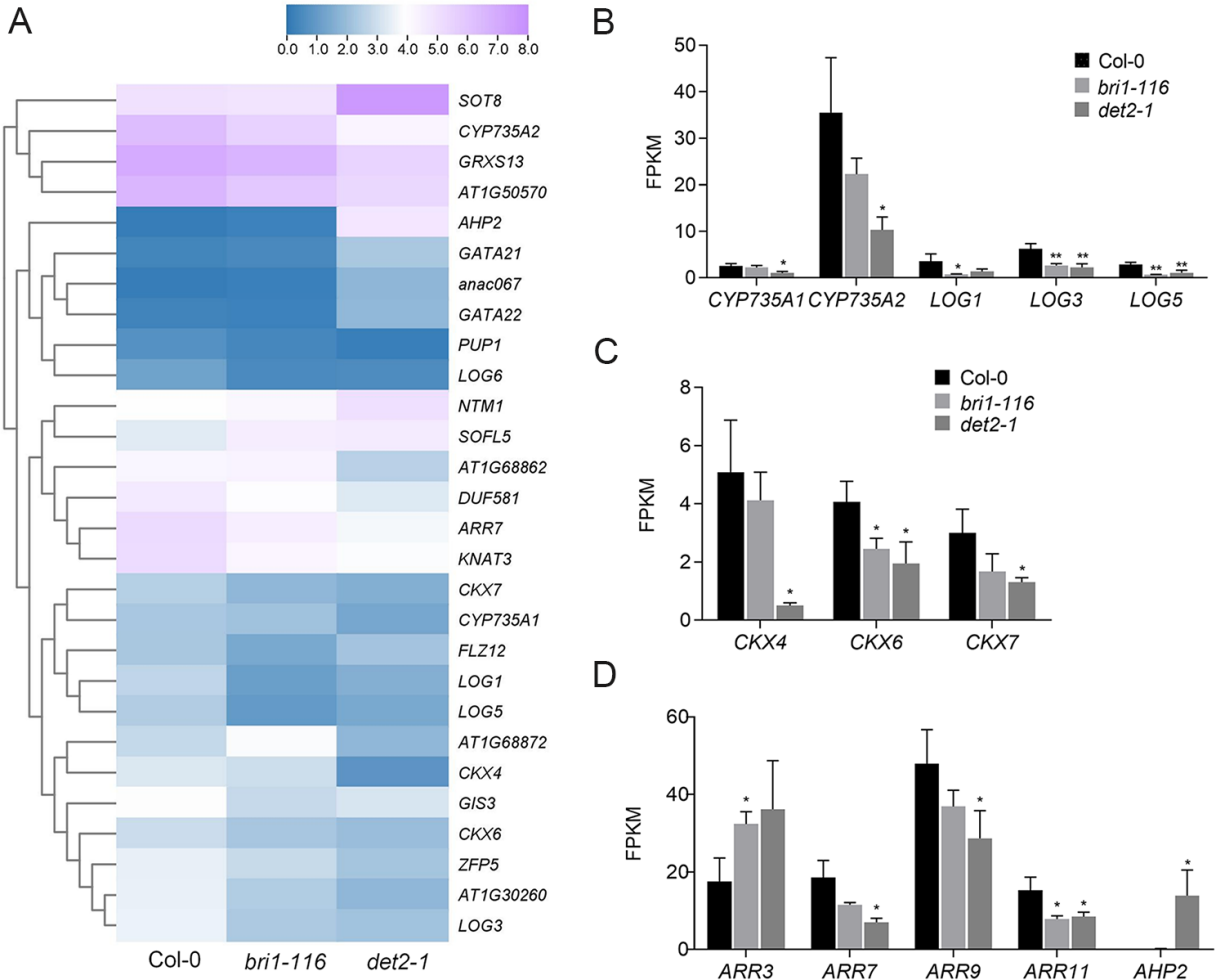
Cytokinin-associated DEGs (**A**) Relative heat maps of RNA-seq. Rows represent cytokinin-related genes, columns represent Col-0, *bri1-116* and *det2-1*. Blue and purple boxes represent genes showing lower and higher expression levels, respectively. Color saturation reflects the magnitude of the expression level for each gene. (**B-D**) Comparison of the cytokinin-related gene expression levels (by FPKM values) in Col-0, *bri1-116* and det2-1. *P < 0.05, **P < 0.01 (t-test)

Several DEGs associated with cytokinin signaling were also detected by our RNA-seq data. Expression of cytochrome P450 monooxygenase *CYP735A1* and *CYP735A2* was significantly decreased in *det2-1* mutants, and the lysine decarboxylase family protein *LOG1*, *LOG3*, and *LOG5* and cytokinin oxidase *CKX4*, *CKX6*, and *CKX7* were down-regulated in *det2-1* mutants. Expression of some cytokinin response regulatory factors ARR also changed to varying degrees. *ARR3* was up-regulated in *bri1-116* while *ARR7*, *ARR9*, and *ARR11* were down-regulated in *det2-1*. Similarly, the phosphate transporter gene *AHP2* in *det2-1* mutants was also up-regulated (Fig. 5).

### Identification of GA-related DEGs

**Fig. 6:**
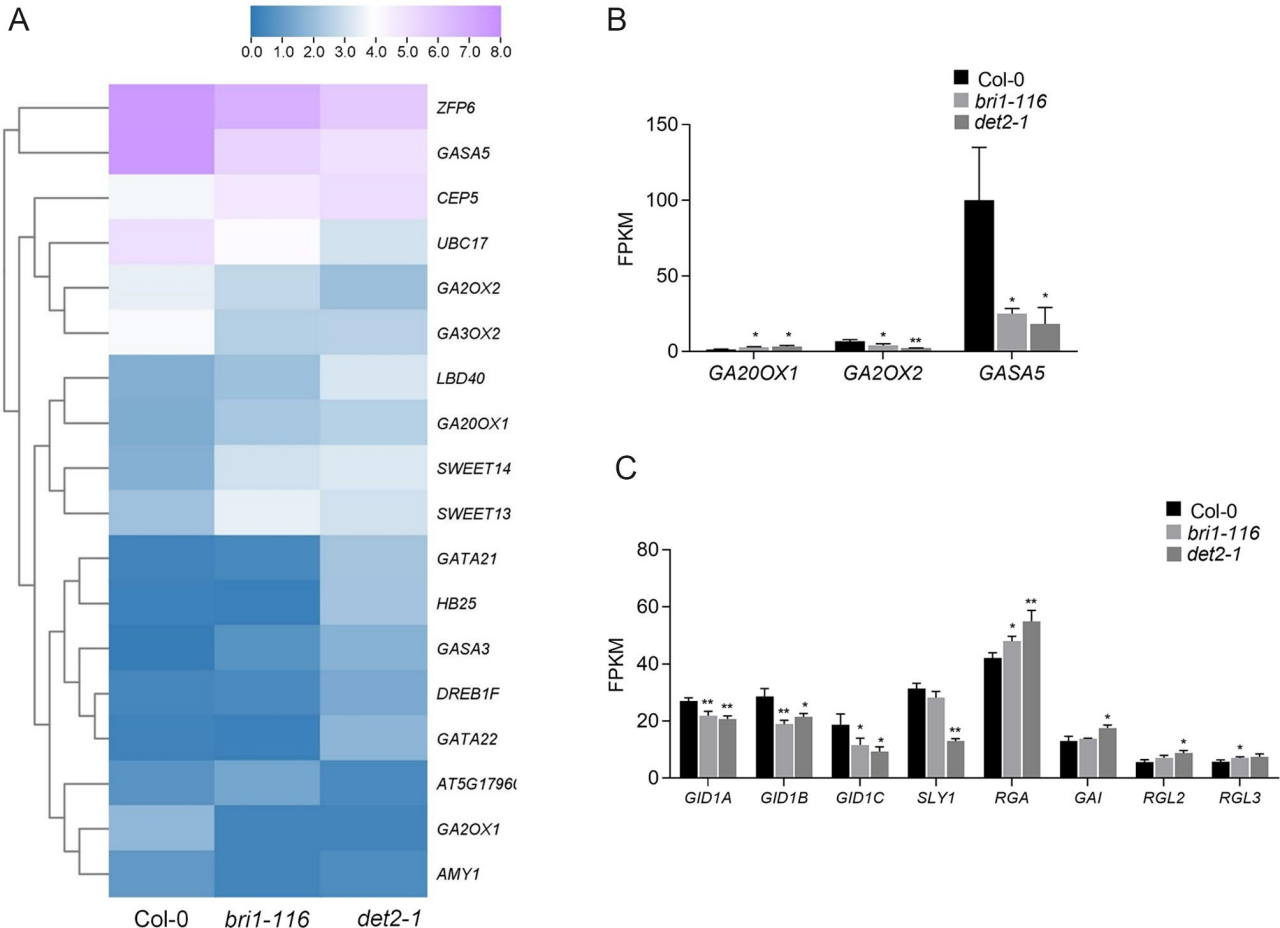
GA-associated DEGs (**A**) Relative heat maps of RNA-seq. Rows represent GA-related genes, columns represent Col-0, *bri1-116* and *det2-1*. Blue and purple boxes represent genes showing lower and higher expression levels, respectively. Color saturation reflects the magnitude of the expression level for each gene. (**B-C**) Comparison of the GA-related gene expression levels (by FPKM values) in Col-0, *bri1-116* and det2-1. *P < 0.05, **P < 0.01 (t-test)

Among DEGs related to GA, *GA20OX1* was up-regulated and *GA2OX2* was down-regulated in both mutants. Similarly, the *GID1* and *SLY1* were down-regulated while the DELLA protein family members, *RGA*, *GAI*, *RGL2*, and *RGL3* were up-regulated to varying degrees. Moreover, in both mutants, the GA-stimulated gene *GASA5* was down regulated in both mutant (Fig. 6).

### Identification of jasmonates-related DEGs

**Fig. 7:**
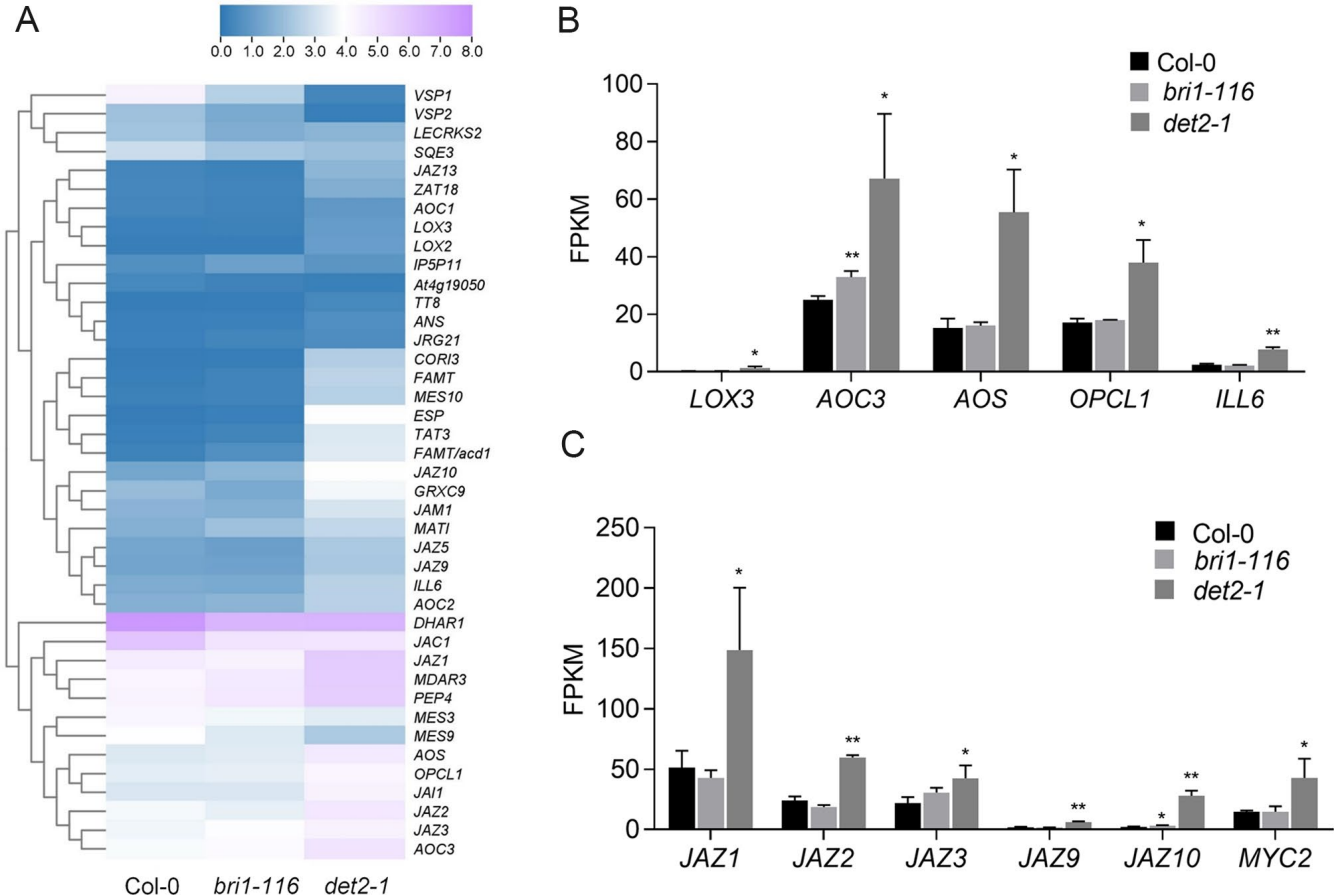
JA-associated DEGs (**A**) Relative heat maps of RNA-seq. Rows represent JA-related genes, columns represent Col-0, *bri1-116*, and *det2-1*. Blue and purple boxes represent genes showing lower and higher expression levels, respectively. Color saturation reflects the magnitude of the expression level for each gene. (**B-C**) Comparison of the JA-related gene expression levels (by FPKM values) in Col-0, *bri1-116* and det2-1. *P < 0.05, **P < 0.01 (t-test)

JA is a kind of plant endogenous hormone, that exists widely in plants and plays an important role in plant growth and development. Our transcriptome data revealed that more JA-associated DEGs were found in *det2-1* mutants than in *bri1-116* mutants. Enzymes related to JA biosynthesis, such as lipoxygenase *LOX3*, allene oxide cyclase *AOC3*, allene oxide synthase *AOS* and CoA ligase *OPCL1*, were significantly upregulated in the *det2-1* mutant. An amidohydrolase *ILL6* which contributed to JA-Ile turnover was also significantly up-regulated in the *det2-1* mutant. A number of transcription repressor *JAZ* (jasmonate ZIM-domain protein) family genes were detected and were up-regulated in *det2-1* mutants (Fig. 7). In addition, the transcription activator *MYC2* was also significantly upregulated in *det2-1* mutants (Fig. 7). We then verified the expression pattern of JA synthase *AOC* and *AOS*, and catalytic enzyme *ILL6* in both *det2-1* and *bri1-116* mutants through qPCR experimental analysis and found that the results were consistent with the RNA-Seq results. Unlike *bri1-116*, these genes were significantly up-regulated in *det2-1* mutant (Figure S5), suggesting that DET2 may be involved in regulating JA signaling in Arabidopsis.

## Discussion

Unlike animals, plants are sensible. Thus, they have evolved a strong ability to sense the surrounding stimulus, and have to respond accordingly to ever-changing environments and challenges. Gravity is an important environmental factor affecting plant growth and is one of the main factors determining plant root configuration. BRs are important regulators of plant growth and development, and when applied exogenously, the eBL can increase Arabidopsis gravitropic curvature [47, 48]. We found that BR synthetic mutants *det2-1* and BR insensitive receptor mutants *bri1-116* had different degrees of root gravitropism loss, indicating that BR is involved in root gravitropism (Fig. 1). Furthermore, auxin plays an important role in plant gravity regulation. Therefore, we studied auxin-related gene changes in *bri1-116* and *det2-1*. BR and auxin had a synergistic effect on gravitropism regulation. Comparing the transcripts abundance of auxin-related genes indicated that auxin and BRs had a mutual regulating effect on Arabidopsis root development and metabolism. Auxin content may be reduced in BR deletion mutants because *GH3.5* which catalyze auxin formation of conjugates, and methyltransferase1 (IAMT1), which catalyzes IAA methylation, are significantly downregulated in *bri1-116* and *det2-1* mutants. Small auxin upregulated RNA (SAUR) genes are a group of early auxin-responsive genes that play a crucial role in plant growth and environmental stimuli. Our transcriptome data showed that many SAUR genes such as *SAUR32*, *SAUR31*, and *SAUR55* were also significantly down-regulated in both *det2-1* and *bri1-116* mutants (Fig. 4). Roots gravity is inseparable from the polar auxin transport, and many specific transporters such as auxin influx (LAX) and auxin outflow (PIN) are related to auxin polar transport, and their expression controls plants auxin balance. We found that several PIN and AUX genes were down-regulated in *bri1-116* and *det2-1* mutants, indicating that the auxin distribution in these two BR mutants may also be altered. Two interacting protein families, ARF and Aux/IAA, are important regulators of auxin-induced gene expression, and are sensitive to auxin. Expression levels of many *ARF* and *Aux/IAA* genes were significantly changed in both BR mutants, which may reflect the endogenous IAA levels in the roots of both BR mutants.

Cytokinin is also involved in root gravitropism regulation [37, 58, 59]. Cytokinins are mainly produced by root cap statocytes and can rapidly change into an asymmetrical activation pattern after gravistimulation, and initiate root bending [60]. Therefore, we analyzed the expression changes of cytokinin signaling related genes in both *bri1-116* and *det2-1* mutants. We found that the expressions of cytochrome P450 monooxygenases *CYP735A1* and *CYP735A2*, which convert iP nucleotide forms to tZT nucleotide forms, phosphoribosehydrolases *LOG1*, *LOG3* and *LOG5*, which convert cytokinins nucleotide forms to free radical forms, and cytokinin oxidase *CKX4*, *CKX6* and *CKX7* significantly decreased in *bri1-116* and *det2*-1 mutants, suggesting that cytokinin levels in BR-deletion mutants may also be reduced (Fig. 5B, C) [61–63]. ARRs, Arabidopsis response regulators, are downstream cytokinin signaling pathways regulators that are activated by receiving phosphate groups from cytokinin receptors. In Arabidopsis, three type-A response regulators, *ARR3*, *ARR7*, and *ARR9*, and one type-B response regulator, *ARR11*, were down regulated in both BR mutants [64]. Up-regulation of cytokinin receptor *AHP2* suggested a possible weakened cytokinin signal transduction in *det2-1* mutants (Fig. 5D).

Gibberellin also showed asymmetric distribution in plant gravitropism. *GA20OX* expression is negatively regulated by active GA feedback, while *GA2OX* expression is feedforward regulated by active GA [65]. We also analyzed the changes of gibberellin-related genes in *bri1-116* and *det2-1*, and found that *GA20OX1* expression was significantly increased, while *GA2OX2* was significantly downregulated in both mutants, suggesting that the gibberellin content in BR deletion mutants may also be reduced (Fig. 6).

Compared with *bri1-116*, the *det2-1* mutant showed a stronger gravitropism loss. When seeded on the 1/2 MS medium, the root of *det2-1* mutants tended to grow upwards and exhibit longer, denser root hairs. Our transcriptome data analysis showed that the gene changes related to auxin, gibberellin, cytokinin, and other hormones in *bri1-116* and *det2-1* were basically consistent (Fig. 3B). The difference is that in the *det2-1*, genes associated with the JA signaling pathway appear to have more significant changes. The JA biosynthetic pathway was regulated by positive feedback. JA biosynthesis genes, such as, the expression level of *LOX3*, *AOC3*, *AOS*, and *OPCL* were significantly increased in *det2-1* mutants, may resulting in elevated JA level in *det2-1* mutants (Fig. 7) [66]. JAZ is an important jasmine signal-induced gene expression regulator, which can interact with a series of transcription factors or signal transduction proteins to inhibit plant JA response. JA treatment can cause JAZ protein degradation, which in turn activates the JA corresponding gene. The expression of *JAZ* is also induced by JA, indicating that it may be moderated by negative feedback from JA signaling [67, 68]. We found that a large number of JAZ gene families were upregulated in the *det2-1* mutant, which may reflect the increased levels of endogenous JA.

To explain the root gravitropism change mechanism of BR mutants *bri1-116* and *det2-1*, independent cDNA libraries from the root of these two mutants were constructed and sequenced. A large number of DEGs were identified by transcription analysis. The expression of genes related to auxin, cytokinin, and GA pathways changed significantly in these two mutants while the JA signaling pathway seemed specially associated with *det2-1* mutants, suggesting that DET2 may be involved in more metabolic pathways besides BRs.

## Conclusion

*det2-1* is the BR signaling defective mutant similar to *bri1-116* but shows differences in some characteristics, such as partial loss of the root gravity. We systematically compared the transcriptome of roots in *det2-1* and *bri1-116* mutants and found significant differences in gene expression between these two. We found that the genes related to various hormones in *bri1-116* and *det2-1* were changed, but only the expression levels of JA-related genes were significantly different in *det2-1* and *bri1-116*. Therefore, it is speculated that the changes in JA signal may be the reason for the gravity loss in *det2-1*. Due to the complexity of the detection method of JA, we used transcriptome analysis to provide new insight for subsequent studies on DET2 gene in root gravity.

### Electronic supplementary material

Below is the link to the electronic supplementary material.

Supplementary Material 1

Supplementary Material 2

Supplementary Material 3

Supplementary Material 4

Supplementary Material 5

## Data Availability

The data or material of this study are available from the corresponding author, H.Y.R., upon reasonable request. The raw sequencing files of the transcriptome data are now available in NCBI SRA database with the following BioProject ID, PRJNA1112142 (http://www.ncbi.nlm.nih.gov/bioproject/1112142), and the SRA accessions are SRR29049046, SRR29049043, SRR29049044, SRR29049041, SRR29049038, SRR29049045, SRR29049042, SRR29049039, SRR29049040.
