# Supplementary material for: Molecular mechanism of brassinosteroids involved in root gravity response based on transcriptome analysis
Source: BMC Plant Biol. 2024 Jun 1;24:485. doi: 10.1186/s12870-024-05174-6 (PMC11143716; doi:10.1186/s12870-024-05174-6)
Supplement: Supplementary file 1 — Supplementary Material 1 [file 12870_2024_5174_MOESM1_ESM.pdf]

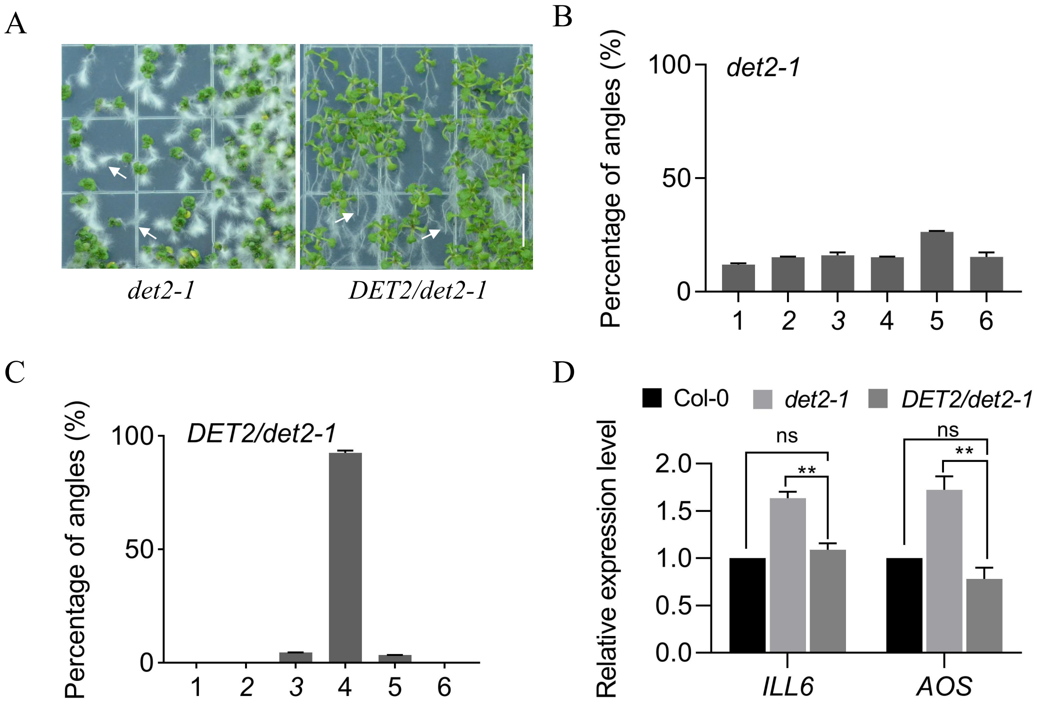

Figure S1. Overexpression of *DET2* could completly restore less gravity phenotype of *det2-1*. A. The 7-day-old seedings of *det2-1* and *DET2/det2-1* grown vertically in 1/2 MS medium. The white arrow indicates the root of the seedling. Scale bar, 1.5cm. B, C. Distribution of the root gravitropic angle in *det2-1* and *DET2/det2-1* within 6 bins covering 360°. D. qRT-PCR analysis of *ILL6* and *AOS* in transgenic plant, *ACT2* served as an internal control.

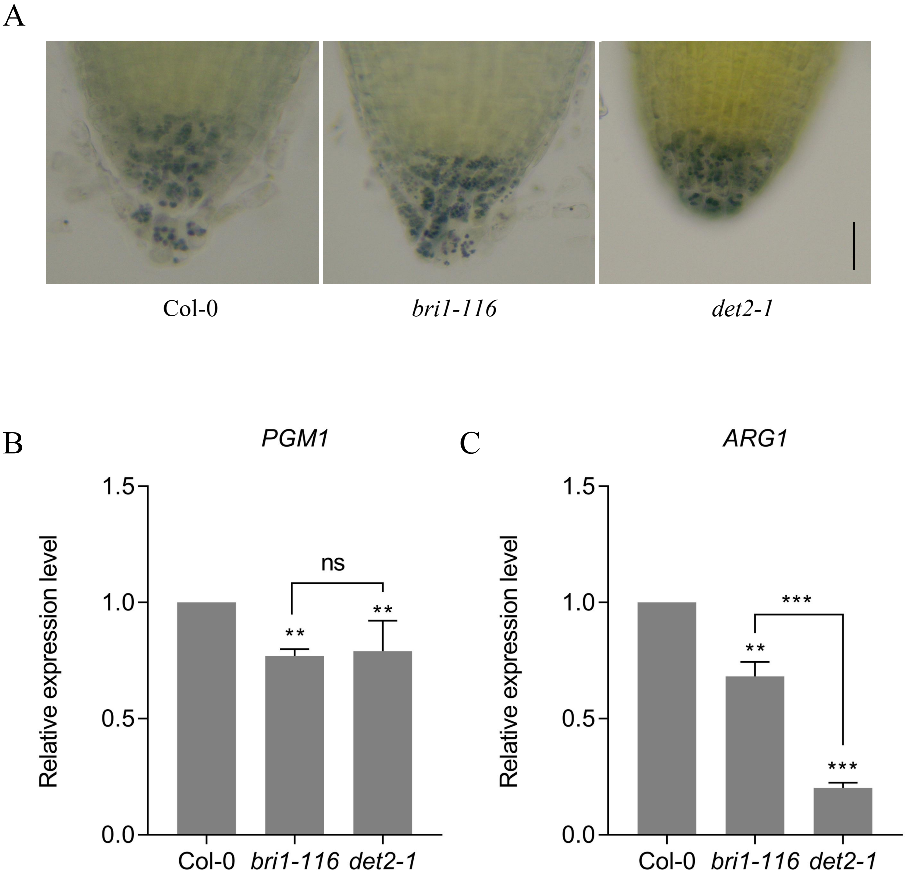

Figure S2. Starch staining and qRT detection for Col-0, *det2-1* and *bri1-116* Lodine staining. A. Starch accumulation in the root cap of Col-0, *det2-1* and *bri1-116.* B, C. qRT-PCR analysis of *PGM1* and *ARG1* in root, *ACT2* served as an internal control.

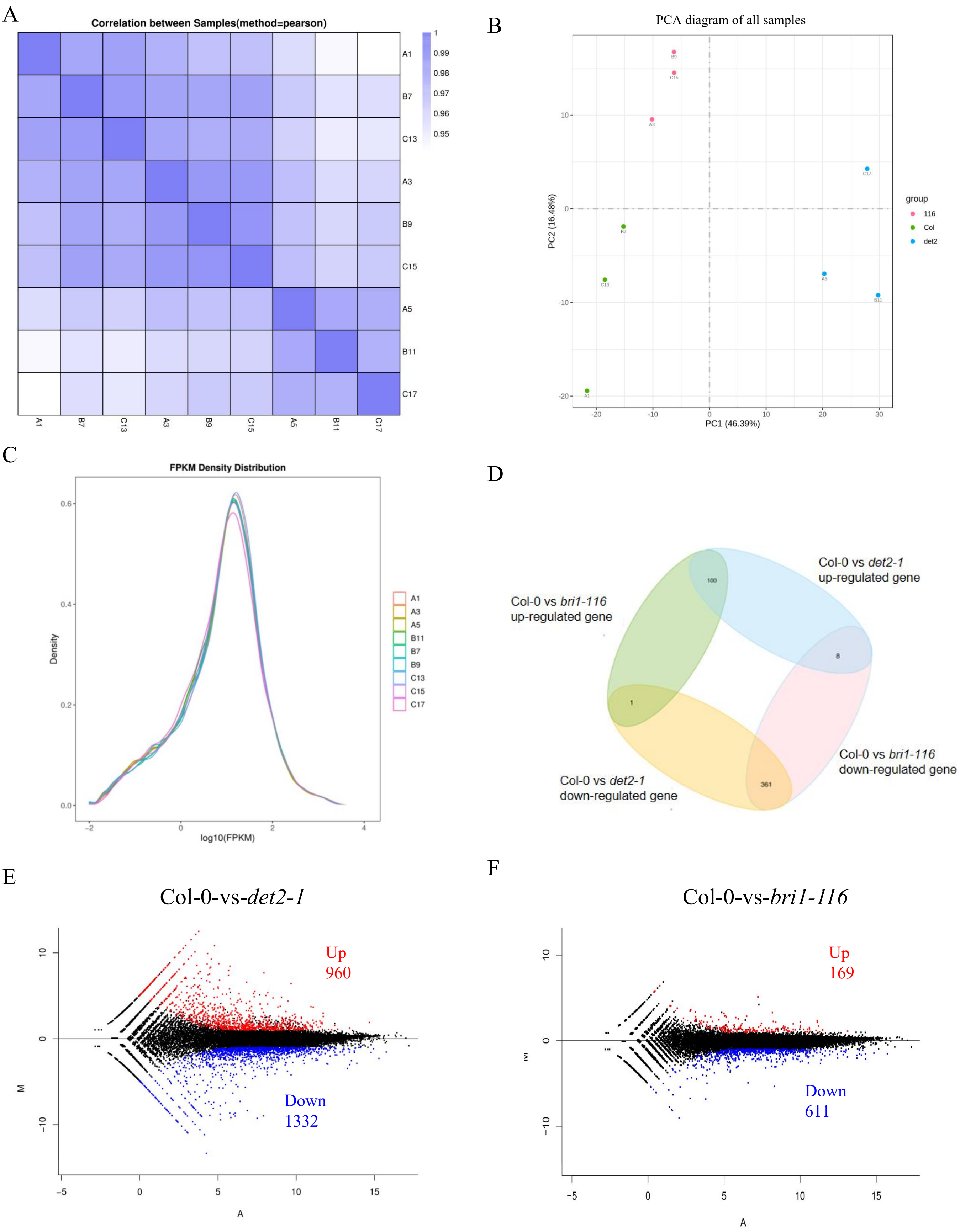

Figure S3. RNA seq quality evaluation analysis and MA plot and Venn diagram of DEGs. A. Correlation heat map of gene expression levels of all samples. B. The principal component analysis (PCA) diagram of all samples. C. The FPKM density distribution of genes in different samples. D. Venn diagram of DEGs. E. The MA plot of DEGs of Col-0-vs-*bri1-116*. F. The MA plot of DEGs of Col-0-vs-*det2-1*. The black, red and blue dot represented the number of genes that expressed insignificantly, up-regulated and down-regulated significantly, respectively.

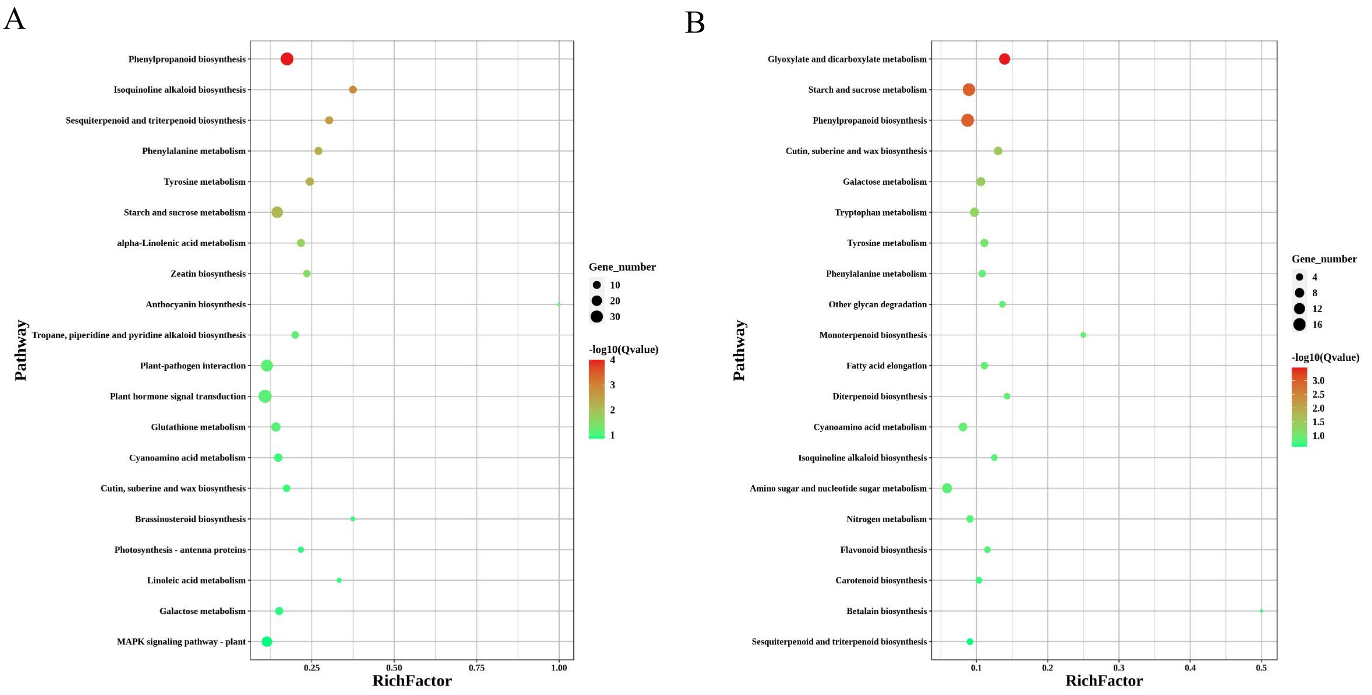

Figure S4. KEGG pathways for the top 20 of DEGs of *det2-1* and *bri1-116*. A. KEGG analysis on *det2-1* mutant. B. KEGG analysis on *bri1-116* mutant.

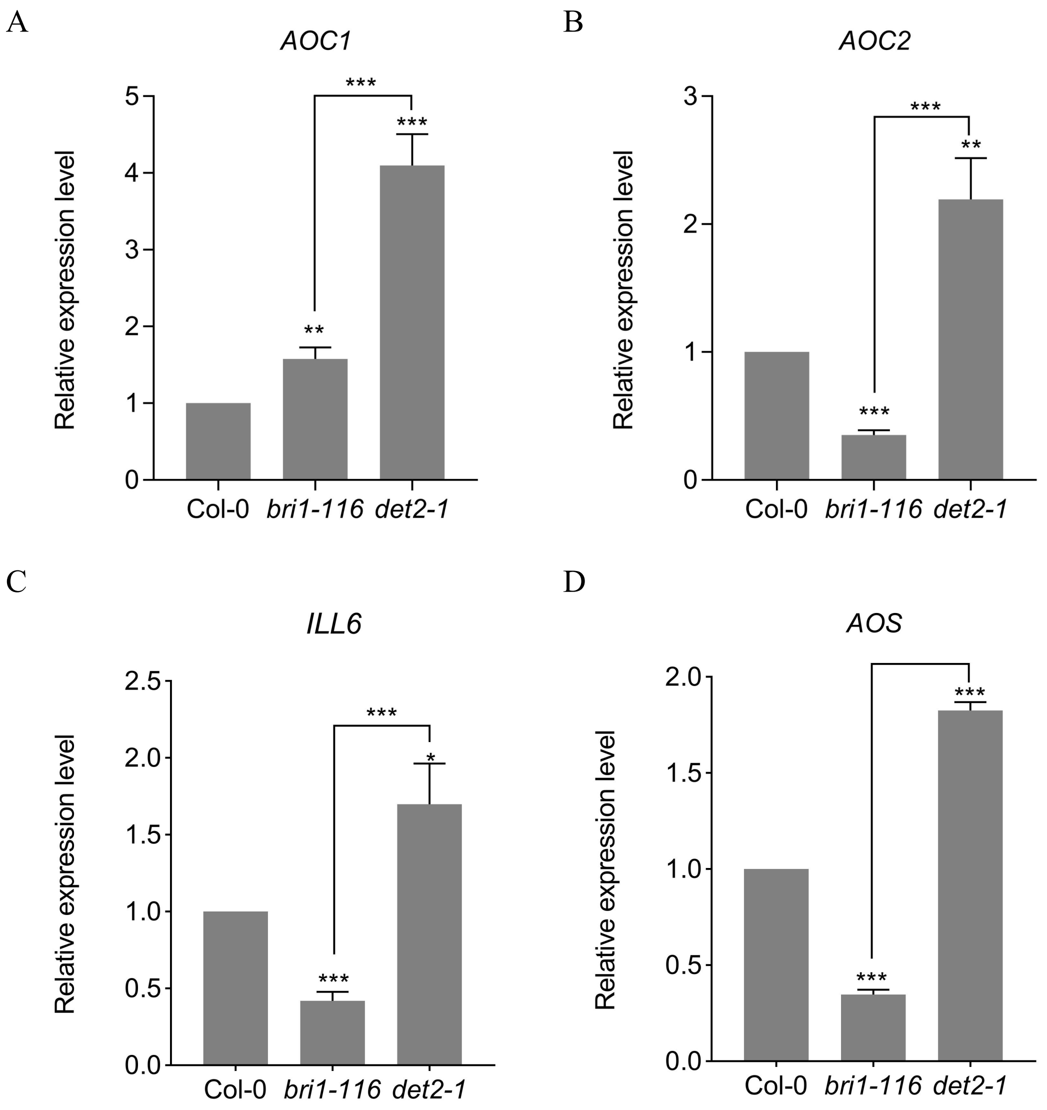

Figure S5. qRT detection for Col-0, *det2-1* and *bri1-116*. A-D. qRT-PCR analysis of *AOC1* , *AOC2* , *ILL6* and *AOS* in root, *ACT2* served as an internal control.
